# Genome assembly of *Hibiscus sabdariffa* L. provides insights into metabolisms of medicinal natural products

**DOI:** 10.1093/g3journal/jkae134

**Published:** 2024-07-12

**Authors:** Taein Kim, Jeong Hun Lee, Hyo Hyun Seo, Sang Hyun Moh, Sung Soo Choi, Jun Kim, Sang-Gyu Kim

**Affiliations:** Department of Biological Sciences, KAIST, Yuseong-gu, 34141 Daejeon, Republic of Korea; Plant Cell Research Institute, BIO-FD&C Co., Ltd, Yeonsu-gu, 21990 Incheon, Republic of Korea; Plant Cell Research Institute, BIO-FD&C Co., Ltd, Yeonsu-gu, 21990 Incheon, Republic of Korea; Plant Cell Research Institute, BIO-FD&C Co., Ltd, Yeonsu-gu, 21990 Incheon, Republic of Korea; Daesang Holdings, Jung-gu, 04513 Seoul, Republic of Korea; Department of Convergent Bioscience and Informatics, College of Bioscience and Biotechnology, Chungnam National University, Yuseong-gu, 34134 Daejeon, Republic of Korea; Department of Biological Sciences, KAIST, Yuseong-gu, 34141 Daejeon, Republic of Korea

**Keywords:** biosynthetic pathway, HiFi sequencing, *Hibiscus sabdariffa*, natural product

## Abstract

*Hibiscus sabdariffa* L. is a widely cultivated herbaceous plant with diverse applications in food, tea, fiber, and medicine. In this study, we present a high-quality genome assembly of *H. sabdariffa* using more than 33 Gb of high-fidelity (HiFi) long-read sequencing data, corresponding to ∼20× depth of the genome. We obtained 3 genome assemblies of *H. sabdariffa*: 1 primary and 2 partially haplotype-resolved genome assemblies. These genome assemblies exhibit N50 contig lengths of 26.25, 11.96, and 14.50 Mb, with genome coverage of 141.3, 86.0, and 88.6%, respectively. We also utilized 26 Gb of total RNA sequencing data to predict 154k, 79k, and 87k genes in the respective assemblies. The completeness of the primary genome assembly and its predicted genes was confirmed by the benchmarking universal single-copy ortholog analysis with a completeness rate of 99.3%. Based on our high-quality genomic resources, we constructed genetic networks for phenylpropanoid and flavonoid metabolism and identified candidate biosynthetic genes, which are responsible for producing key intermediates of roselle-specific medicinal natural products. Our comprehensive genomic and functional analysis opens avenues for further exploration and application of valuable natural products in *H. sabdariffa*.

## Introduction

*Hibiscus*, a popular genus in the Malvaceae family, contains more than 200 species distributed in tropical and subtropical regions. Plants in genus *Hibiscus* are widely known to produce valuable natural products such as fibers, pigments, and medicinal secondary metabolites. As a medicinal plant, the phytochemical and pharmacological properties of *Hibiscus sabdariffa* have been well studied (Da-Costa-Rocha *et al*. 2014). The calyx of *H. sabdariffa* contains various bioactive organic acids, including phenolic acid, hydroxycitric acid, and hibiscus acid. In addition, *H. sabdariffa* contains anthocyanins and flavonoids such as delphinidin, cyanidin, hibiscetin, gossypetin, and quercetin (Da-Costa-Rocha *et al*. 2014). These bioactive compounds have been the subject of considerable research interest due to their potential health benefits and therapeutic properties. In *H. sabdariffa* extract, quercetin and its derivative, quercetin-3-*O*-glucuronide, have been found to reduce the levels of reactive oxygen species (ROS) induced by glucolipotoxicity. They also reduce triglyceride accumulation, which is critical for antiobesity (Herranz-López *et al*. 2020). In addition, the roselle extracts showed antihypertensive, anti-inflammatory, and antimicrobial effects (Herrera-Arellano *et al*. 2004; Mardiah *et al*. 2015; Abdallah 2016).

Revealing the biosynthetic pathways of key natural products responsible for these medicinal effects would facilitate the pharmacological application of *H. sabdariffa*. Recently, transcriptomics-based approaches have been developed as effective tools for revealing the biosynthesis of complex natural products; biosynthesis of colchicine, benzylacetone, catharanthine, and olive sesquiterpenes were recently identified based on RNA-seq coexpression analysis (Nett *et al*. 2020; Kang *et al*. 2022; Stander *et al*. 2022; Sun *et al*. 2022b; Alagna *et al*. 2023; Li *et al*. 2023). Reliable transcriptome analysis and manipulation of gene expression require high-quality genomic resources from the host organism (Hibrand Saint-Oyant *et al*. 2018; Raymond *et al*. 2018; Sun *et al*. 2022b; Li *et al*. 2023). Moreover, comparative genomics studies also need high-quality genomic resources, which provide critical evidence for the biogenesis of novel natural products. These evidences offer insights into evolutionary aspects and functional diversification (Zhang *et al*. 2020, 2023; Tang *et al*. 2022; Sun *et al*. 2022a; Conart *et al*. 2023; Wang *et al*. 2023).

High-quality genomes of *Hibiscus mutabilis*, *Hibiscus syriacus*, and *Hibiscus trionum* have been published (Li *et al*. 2015; Kim *et al*. 2017; Yang *et al*. 2022; Koshimizu *et al*. 2023), and these genomes have built the fundamental basis for the genomic approaches. However, the available genome for *H. sabdariffa* is currently limited to a poor-quality draft (JAPQLB000000000.1, GenBank), which poses a challenge for comprehensive genomic and biochemical studies in roselle (Kim *et al*. 2017; Yang *et al*. 2022; Koshimizu *et al*. 2023). Recently, significant progress has been made in long-read sequencing technologies, particularly with the introduction of high-fidelity (HiFi) sequencing by Pacific Biosciences. This has led to even greater accuracy in long-read sequencing compared to traditional Illumina short-read sequencing. Leveraging this advanced technology has the potential to significantly improve the quality of genomic resources for *H. sabdariffa.*

In this study, we report high-quality genomic resources of *H. sabdariffa*. We obtained reliable genome assemblies and their gene models of *H*. *sabdariffa* by applying the state-of-the-art HiFi long-read DNA sequencing technology and short-read RNA sequencing technology. For further usage of other researchers, we evaluated the quality of genomes and annotated genes. We utilized these genomic resources to identify genes involved in natural product biosynthetic pathways in roselle. Our high-quality genomic resources and functional analysis provide opportunities for biochemical understanding of *H. sabdariffa* and its natural products.

## Materials and methods

### Plant materials

Commercially available *H*. *sabdariffa* seeds (Cheonnyangssiat, Chungcheongbuk-do, South Korea) originated from Hong Kong were germinated and grown in a plant growth room of 16-h light and 8-h dark photoperiod with controlled temperature at 25 ± 2°C and moiety at 60%.

### DNA/RNA sequencing

The genomic DNA of *H. sabdariffa* plant was extracted from mature leaves and sequenced by NICEM (Seoul National University, South Korea) using the following protocol. The extraction method employed was based on the cetyltrimethylammonium bromide (CTAB) method, known for its high-molecular-weight DNA extraction capabilities (Porebski *et al*. 1997). The extraction procedure involved cell lysis of leaf tissues frozen on liquid nitrogen, DNA purification by the use of polyvinyl pyrrolidone, DNA concentration by vacuum-dry, and sequential elimination of short DNA to ensure the quality and integrity of the extracted genomic DNA. Extracted genomic DNA was qualified with NanoDrop, Quantus, and agarose gel electrophoresis to validate whether its quality is adequate to be sequenced. The PacBio HiFi sequencing libraries were prepared with SMRTbell prep kit 3.0 (Pacific Biosciences, Menlo Park, USA) and were sequenced on the SMRTbell barcoded adapter plate (Pacific Biosciences) on the PacBio Sequel IIe platform (Pacific Bioscience).

Total RNA extraction from mature leaves and RNA sequencing were conducted by Macrogen (Seoul, South Korea). Total RNA was extracted with the RNeasy Plant Mini Kit (Qiagen, Hilden, Germany), and RNase-free DNase I (Qiagen) was used to eliminate its genomic DNA contamination. Quality of RNA was measured with the RNA integrity number score on the Agilent 2100 Bioanalyzer (Agilent Technologies, Santa Clara, USA). The RNA sequencing libraries were generated with the TruSeq Stranded Total RNA with the Ribo-Zero Plant kit (Illumina, San Diego, USA) and were sequenced on the NovaSeq 6000 system (Illumina).

### De novo genome assembly

Genome assembly and gene annotation were performed following a protocol paper (Kim and Kim 2022). In general, we used the programming languages R (v4.3.2) and Python (v.3.9.10) and the virtual environment Anaconda3 (v22.9.0) for the following programs. Our HiFi reads were de novo assembled into contigs using the Hifiasm assembler (v0.18.5-r500, cloned from official GitHub repository on 2023 February 14) on defaults options except *-s 0.54* (*-s* represents the similarity threshold). It produced 3 main genome assemblies: a primary genome assembly that contains a complete assembly and 2 partially phased, haplotype-resolved genome assemblies, that is, haplotigs (Hap 1 and Hap 2 in this paper). The resulting graphical fragment assembly (GFA)-formatted genome assembly files were converted into FASTA-formatted files. The primary assembly and the 2 haplotigs were treated individually for all the following analysis.

### Repeat and gene annotation

Since there is no available specific repeat library for *H. sabdariffa*, a de novo repeat library of *H. sabdariffa* was constructed using RepeatModeler (v2.0.3) with NINJA (NINJA-0.95-cluster_only; Flynn *et al*. 2020). This library and the known repetitive sequences in a predefined eudicot repeat library were used for RepeatMasker (v4.1.2) to mask repetitive regions of our genome assemblies with default options (Saha *et al*. 2008). As RepeatMasker predicts repeat regions based on classified repeat libraries and leverages repeat classification models internally, the results of repeat masking by RepeatMasker contained transposable element classification. Hard-masked outputs were utilized for analysis of repetitive regions, and soft-masked outputs were further utilized for gene annotation.

Based on repeat masking results, we found the telomeric repeats in the genome applying “grep” command to find the plant canonical telomeric repeat sequence “TTTAGGG” and its variant forms (Richards and Ausubel 1988; Adamusová *et al*. 2020). The candidate telomeric regions were inferred by the position and length, i.e. the telomeric repeats placing in the end of contig and longer than 1,000 bp (i.e. ∼140 concatemers of telomeric repeat) were considered as candidate telomeres. When the position of telomeric repeats is 5′ end, the sequence should be “CCCTAAA” and its variant forms, and “TTTAGGG” and its variant forms were required for 3′ end.

Total RNA sequencing reads were aligned to the soft-masked genomes with HISAT2 (v2.2.1), and the alignment results were sorted into the binary alignment and map (BAM) format with SAMtools (v1.15 using htslib 1.14; Li *et al*. 2009; Kim *et al*. 2019). Based on the RNA-seq alignment results and ab initio gene prediction model, genes were annotated for each soft-masked genome with Braker2 (v2.1.6 using Genemark-ES/ET/EP v.4.71_lic) default options (Stanke *et al*. 2006, 2008; Li *et al*. 2009; Barnett *et al*. 2011; Buchfink *et al*. 2014; Lomsadze *et al*. 2014; Hoff *et al*. 2016; Brůna *et al*. 2020, 2021). The Braker2 pipeline incorporates an Augustus algorithm that has been trained by RNA-seq alignment and protein alignment for ab initio gene prediction (Brůna *et al*. 2021).

### Evaluation

Qualities of assembled genome and annotated genes were evaluated based on length statistics and benchmarking universal single-copy ortholog (BUSCO) results (Simão *et al*. 2015). BUSCO (v.5.2.2) analysis was performed on the eudicots_odb10 database to confirm whether assembled genomes and predicted gene sets appropriately contain the evolutionary conserved genes (Simão *et al*. 2015). The results of BUSCO analysis were plotted by generate_plot.py script contained in a BUSCO package. We employed the assembly-stats (v1.0.1) to analyze the length statistics of contigs and genes, including the number, mean length, maximum length, N50 value, and total size (Sjunnebo 2007). The consensus quality (QV) of each genome was assessed by using meryl (v1.3) and Merqury (v1.3; Rhie *et al*. 2020). LTR Assembly Index (LAI) could be used to assess the continuity of genome assembly in a reference-free manner. The LAI of each genome was evaluated by using LTR_retriever (v2.9) with default options described in an official open-source GitHub repository of it: https://github.com/oushujun/LTR_retriever (Ou *et al*. 2018; Ou and Jiang 2018). The gene annotation quality was further assessed with the RNA read alignment rate onto genic region by using BEDTools (v2.27.1). The BAM files generated by aligning the RNA-seq data onto each genome in the *Repeat and gene annotation* section were used for this analysis.

### Identification of whole-genome duplication

For the identification of whole-genome duplication (WGD), we used a synonymous substitution rate (Ks) plot-based method. First, BLASTN (v2.10.0+) was employed to perform similarity searches between homologous genes in the *H. sabdariffa* primary genome assembly (Altschul *et al*. 1990). Subsequently, we utilized FASTKs (v2023.2.16) for Ks plot analysis (Suyama *et al*. 2006; Yang 2007; Mckain *et al*. 2016). To enhance the accuracy of our methodology in WGD detection, we further applied Mclust (v6.0.0), a tool specifically tailored for identifying duplicated regions (Fraley and Raftery 2002).

### Structural variants and single nucleotide polymorphism calling

We examined structural variants (SVs) between haplotigs and single nucleotide polymorphisms (SNPs) among primary assembly and haplotigs. Since the Hap 2 showed the longer N50 length than Hap 1, Hap 2 was treated as reference genome in SV calling. Minimap2 (v2.24-r1122) was used to map the Hap 1 assembly to Hap 2, followed by sorting to generate a genome mapping BAM file using SAMtools (Li *et al*. 2009; Li 2018). Based on the genome-to-genome mapping BAM file, svim-asm (v1.0.3) called the SVs between 2 haplotigs, generating a variant call format (VCF) file (Heller and Vingron 2020; Supplementary File 1). By comparing this VCF file to protein-coding regions of the Hap 2 genome using BEDTools (v2.27.1), genes related with SVs were selected (Quinlan and Hall 2010). To call SNPs, raw HiFi reads were mapped to each assembly using minimap2 and SAMtools (v2.24-r1122 and v1.15, respectively; Li *et al*. 2009; Li 2018). DeepVariant-gpu (v1.5.0) was utilized as a SNP caller, producing the VCF result files (Poplin *et al*. 2018). The VCF files were annotated with SnpEff (v5.1d) to predict the effect of SNPs (Cingolani *et al*. 2012) on genes (Supplementary File 1).

### Genetic metabolic network construction

The coding sequences (CDS) of genes predicted in primary and haplotig genomes were functionally annotated by similarity-based sequence search on UniProt Knowledgebase (release 2023.01) using MMseqs2 (v13.4511; Steinegger and Söding 2017; The UniProt Consortium 2017). The major metabolic profile of *H. sabdariffa* was defined based on PubChem taxonomy data (Supplementary Data 1), the referenced compound–organism pair database, LOTUS (Kim *et al*. 2021). To construct genetic metabolic network of the major metabolic profile of roselle, we referred the corresponding molecular pathways of the KEGG database (Kanehisa and Goto 2000). Subsequently, we extracted annotations of biosynthetic enzymes involved in the production of key intermediate compounds from pathways in other organisms using the KEGG database (Kanehisa and Goto 2000). To identify potential candidate genes for biosynthetic pathways in *H. sabdariffa*, we manually compared these annotations of interests to the functional gene annotations of *H. sabdariffa* genes, constructing a gene set potentially involved in the phenylpropanoid and flavonoid pathways. The expression level of each candidate gene was evaluated by aligning the RNA-seq data to Hap 2 genome with kallisto (0.50.1), which was used for gene annotation (Bray *et al*. 2016). We have investigated candidate biosynthetic gene clusters in Hap 2 genome by using plantiSMASH (Kautsar *et al*. 2017). This approach allowed us to narrow down and prioritize the candidate genes for further investigation and characterization in the context of biosynthetic pathways in *H. sabdariffa*.

Following the methods of Lopez-Nieves *et al*. (2018) and Schenck *et al*. (2015), a phylogeny analysis was performed in the TyrA family proteins of *H. sabdariffa*, *H. syriacus*, and *H. trionum*. Candidate genes encoding TyrA family proteins from *Arabidopsis thaliana*, *Beta vulgaris* c. Boltardy, *Spinacia oleracea*, *Nepenthes alata*, *Mirabilis jalapa*, *Rivina humilis*, *Portulaca oleracea*, *Spergularia marina*, *Paronychia polygonifolia*, *Glycine max* c. Williams 82, and *Medicago truncatula* were included in this analysis (Schenck *et al*. 2015; Lopez-Nieves *et al*. 2018). The initial identification of genes encoding TyrA family protein from *H. sabdariffa*, *H. syriacus*, and *H. trionum* involved tBLASTn searches using AtADH1, AtADH2, and GmPDH1 as query protein sequences, executed through Geneious Prime (v11.0.14.1+1). The CDS of all genes were translated into protein sequences, which were then subjected to multiple sequence alignment (MSA) employing Clustal Omega (v1.2.2) in Geneious Prime (v11.0.14.1+1; Sievers *et al*. 2011). The phylogeny of the aligned protein sequences was estimated by PhyML (v3.3.20180621; Guindon *et al*. 2010).

### Comparative genomic analysis

We conducted a comparative analysis of the assembled genome of *H. sabdariffa* with the genomes of taxonomically related organisms. Genome-wide synteny analysis was performed within the *Hibiscus* genus: *H. syriacus*, *H. trionum*, and *H. sabdariffa* (Hap 2). This comparison involved genome-wide synteny analysis based on BUSCO genes, using the eudicots_odb10 database (BUSCO v5.2.2). BUSCO analysis was conducted for each organism individually to identify shared single-ortholog genes using awk and grep. The position of each BUSCO gene across the genomes was visually represented with Circos (v0.69-8; Krzywinski *et al*. 2009). Genomes of Malvaceae species and *A. thaliana* from the GenBank database were collected for the phylogenomic analysis. A BUSCO analysis was carried out on all genomes, and targets for phylogenomic analysis were selected based on 2 criteria: a complete BUSCO score above 90% and a higher count of single-copy BUSCOs in comparison to duplicated BUSCOs. Subsequent to the BUSCO analysis, 436 shared single-copy orthologs were identified within the target genomes, and these orthologs were concatenated for each genome, which led to the formation of 19 queries. MSA was carried out on 19 queries using MAFFT (v7.522), with the max_iteration option set to 1,000 (Katoh and Standley 2013). Subsequently, the MSA results were utilized to construct a phylogenetic tree using RAxML (v1.2.0) with the LG + G8 + F model and 200 bootstraps, as outlined by Stamatakis, (2014). The best tree generated by RAxML was visualized using MEGA (v11.0.13), following the procedure described by Tamura *et al*. (2021). The list of species and their corresponding genome accessions is as follows: *Corchorus capsularis* (GCA_001974805.1), *Corchorus olitorius* (GCA_001974825.2), *Gossypium australe* (GCA_005393395.2), *Gossypium trilobum* (GCA_013467465.1), *Gossypium raimondii* (GCA_013467475.1), *Gossypium lobatum* (GCA_013467485.1), *Gossypium gossypioides* (GCA_013467495.1), *Gossypium aridum* (GCA_013487665.1), *Gossypium laxum* (GCA_013511315.1), *Gossypium klotzschianum* (GCA_013677235.1), *Gossypium davidsonii* (GCA_013677245.1), *Gossypium harknessii* (GCA_013677255.1), *Gossypium armourianum* (GCA_013677265.1), *Gossypium schwendimanii* (GCA_013677275.1), *Gossypium anomalum* (GCA_019455425.1), *Gossypium stocksii* (GCA_020496765.1), *H. trionum* (GCA_030270665.1), and *H. sabdariffa* (Hap 2).

## Results and discussion

### Genome assembly and annotation

**Fig. 1.**
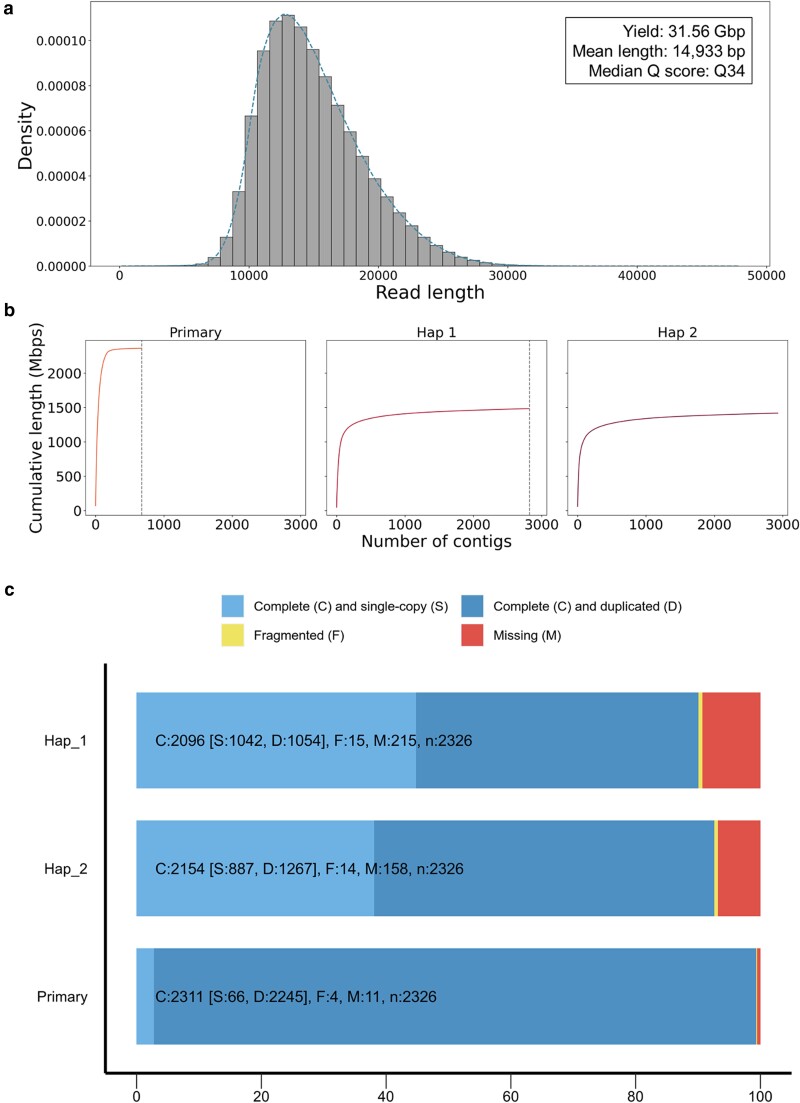
a) The read length distribution of *H. sabdariffa* genomic DNA sequenced by PacBio HiFi sequencing. b) The cumulative length of contigs of haplotypes in descending order. Dotted lines represent the numbers of contigs in primary assembly and Hap 1. c) The BUSCO scores of primary assembly, Hap 1, and Hap 2 genomes using eudicots_odb10 database.

PacBio HiFi sequencing of *H. sabdariffa* genomic DNA resulted in 2.3 million reads (total 33.9 Gb); those mean length was 15.0 kb (Table 1 and Fig. 1a). The HiFi sequencing read quality of *H. sabdariffa* genomic DNA was verified by a Phred quality score (*Q* score) of Q34. This high-quality score assures the reliability and confidence of the sequencing data, allowing accurate downstream analyses and interpretations of the genomic information of *H. sabdariffa*. The karyotype of *H. sabdariffa* is 2n = 4x = 72, and the haploid genome size is 1.67 Gb, i.e. we obtained 20.3× coverage raw reads (Mohammad *et al*. 2020). The HiFi reads were de novo assembled with Hifiasm and phased into pseudo-haplotigs, Hap 1 and Hap 2 (Cheng *et al*. 2021). A haplotig represents a set of haplotype-resolved contigs. The primary assembly represented a total length of 2.36 Gb with a contig N50 length of 26.25 Mb and the longest contig measuring 70.47 Mb (Table 1). For the 2 haplotigs, the total length, N50 length, and maximum length of contigs were found to be 1.42 Gb, 11.96 Mb, and 60.86 Mb for Hap 1 and 1.48 Gb, 14.50 Mb, and 48.40 Mb for Hap 2, respectively (Table 1). Compare to the previous draft genome of *H. sabdariffa*, our genome assemblies represented clearly improved metric (Table 1). The coverage of the primary genome, Hap 1 genome, and Hap 2 genome were 141.3, 86.0, and 88.6% of the 1.67-Gb haploid genome. In this study, Hap 2 was used as a reference haplotype of *H. sabdariffa* genome for SV analysis.

**Table 1.** The raw sequencing data and assembled genomes of *H. sabdariffa*.

Long-read genomic DNA sequencing				
Type	Read count	Yield (bp)	Mean length (bp)	Mean *Q* score
PacBio HiFi sequencing	2,269,272	33,887,858,605	14,993	Q34

RNA sequencing				
Type	Read count	Yield (bp)	Mean length (bp)	>Q30
Illumina PE100	263,077,312	26,570,808,512	100	94.8%

Assembled genomes of *H. sabdariffa*				
	Primary	Hap 1	Hap 2	Previous
Number of contigs	675	2,929	2,823	545,027
Maximum contig (Mb)	70.47	60.86	48.4	1.7
Mean contig (Mb)	3.50	0.48	0.53	0.0004
Contig N50 (Mb)	26.25	11.96	14.5	0.16
Total length (Gb)	2.36	1.42	1.48	0.20
RNA alignment rate	92.47%	87.55%	88.43%	70.42%
Consensus quality (QV)	64.5	61.5	61.6	NA
LTR Assembly Index (LAI)	7.24	6.73	7.74	NA

NA, QV and LAI of the previous draft genome of *H. sabdariffa* were not estimated.

We evaluated each genome with respect to the length distribution of contigs and BUSCO score (Simão *et al*. 2015). The cumulative contig length graphs of the assembled genomes exhibited a left-skewed shape (Fig. 1b), indicating a higher proportion of longer contig lengths. The contig N50 length of 26.25 Mb for primary genome (consisted of the 28th contig) was comparable to 46.39 Mb, the arithmetic average of chromosome lengths (a total genome size/the number of chromosomes) of *H. sabdariffa*. The completeness of genome was further evaluated by BUSCO analysis on the eudicots_odb10 database, which represented 99.3% completeness rate for primary genome, 90.1% for Hap 1, and 92.6% for Hap 2 (Fig. 1c), which are comparable with the chromosome-level genomes of other species in *Hibiscus* genus (Simão *et al*. 2015; Yang *et al*. 2022).

The RNA-seq alignment metrics, QV, and LAI were used to assess the quality of genome assemblies. A primary assembly, Hap 1, and Hap 2 represented 97, 87, and 88% of RNA alignment rate, respectively, which were clearly higher than the RNA alignment rate on previous draft genome, 70% (Table 1). QV and LAI were 64.5 and 7.24 for the primary assembly, where 61.5 and 6.73 for Hap 1 and 61.6 and 7.74 for Hap 2, which all demonstrated the high quality of genome assemblies. QV and LAI of the previous draft genome of *H. sabdariffa* were not estimated, so they were represented as NAs in Table 1.

The tetraploid nature of *H. sabdariffa* poses a challenge in de novo genome assembly, occasionally leading to misassemblies. Hifiasm is renowned for its ability to preserve the contiguity of haplotypes during genome assembly as a leading haplotype-resolved assembler (Cheng *et al*. 2021). In this study, we further deciphered both primary assembly and haplotype-resolved assembly. The primary assembly could represent each homologous sequence once and each heterologous “bubble” twice. The primary genome size of *H. sabdariffa* was 2.36 Gb, which is larger than the haplotype genome (1.67 Gb) but smaller than the diploid genome (3.34 Gb) of *H. sabdariffa*. However, the lower BUSCO score observed in the haplotigs compared to the primary assembly might be due to the inherent challenges in resolving a complexity in tetraploid genomes. On the other hand, the genome sizes of Hap 1 and Hap 2 were smaller than the theoretical haploid genome size (1.67 Gb vs 1.42 and 1.48 Gb, respectively). This difference can be explained by the fact that each haplotype is a reduced version of the primary assembly of the 2x genome (n = 2x = 36). To enhance the phasing and contiguity of the *H. sabdariffa* genome, incorporating parental sequencing information and Hi-C data would improve the resolution of haplotypes, leading to a more comprehensive and accurate representation of the genome. It is worthy to mention that despite this limitation, Hifiasm is a viable solution due to its graph-binning strategy and its capacity for constructing haplotype-resolved assemblies (Cheng *et al*. 2021; Sun *et al*. 2022a).

**Fig. 2.**
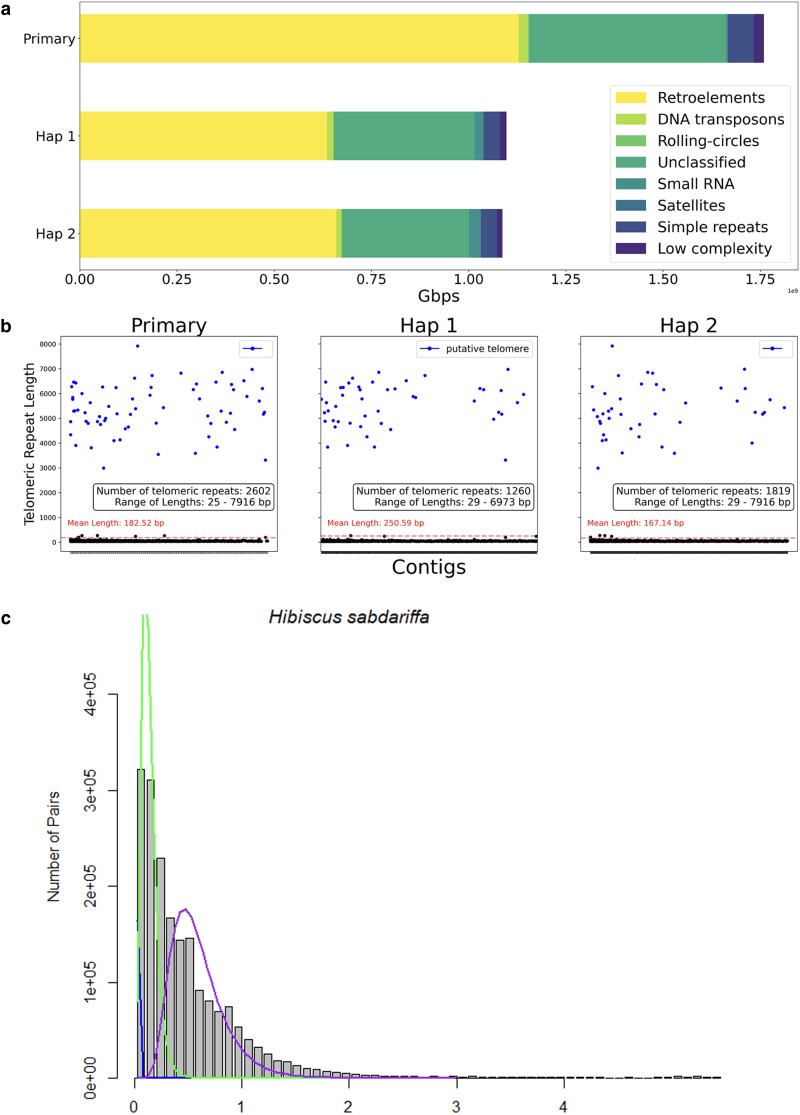
a) The composition of repeat components in *H. sabdariffa* genome. The lengths of repeat components in each genome are described in a bar plot: retroelements, DNA transposons, rolling-circles, unclassified, small RNA, satellites, simple repeats, and low complexity. b) The telomeric repeat distribution in *H. sabdariffa* genome. Dots above the mean length lines represent the candidate telomeres. c) A Ks plot of *H. sabdariffa* primary genome. Three Ks distribution peaks were identified.

We next performed repeat masking with the eudicot repeat library of RepeatMasker. Lower than 4% of genome was annotated as simple and low complexity repeats. On the other hand, after being masked with de novo modeled repeat library, a significant portion of genome was annotated as repeats: 74.48% in the primary genome, 76.67% in Hap 1, and 73.99% in Hap 2 (Fig. 2a and Tables 2 and 3). The Hap 2 genome consists of 42.91% retroelements, 1.10% DNA transposons, and 24.43% unclassified interspersed repeats, where Hap 1 consists of 46.56% retroelements, 0.97% DNA transposons, and 23.05% unclassified interspersed repeats (Table 3). Specifically, more than 40% regions (41.69% in primary assembly, 44.59% in Hap 1, and 41.42% in Hap 2) of genomes were LTR elements, containing Ty1/Copia, Gypsy/DIRS1, and retroviral sequences (Tables 2 and 3).

**Table 2.** Repetitive element annotations of *H. sabdariffa* primary assembly.

Repetitive elements of primary assembly			
Type	Number of elements	Length (bp)	Coverage in genome (%)
Retroelements	923,542	1,128,617,308	47.79
DNA transposons	38,811	25,253,436	1.07
Rolling-circles	1,725	971,658	0.04
Unclassified	1,678,392	507,172,818	21.47
Small RNA	13,660	4,066,571	0.17
Satellites	539	184,424	0.01
Simple repeats	1,014,176	656,886,412	2.83
Low complexity	289,769	26,090,467	1.1
Total	3,960,614	1,759,043,094	74.48

Retroelements			
Type	Number of elements	Length (bp)	Coverage in genome (%)
SINES	932	51,058	0.00
Penelope	3,382	2,160,750	0.09
LINEs	74,279	84,394,825	3.57
LTR elements—Ty1/Copia	424,379	606,739,301	25.69
LTR elements—Gypsy/DIRS1	263,913	377,767,974	16.00
LTR elements—retroviral	82	20,714	0.00

**Table 3.** Repetitive element annotations of *H. sabdariffa* haplotype-resolved genome.

Repetitive elements of Hap 1			
Type	Number of elements	Length (bp)	Coverage in genome (%)
Retroelements	458,524	659,844,473	46.56
DNA transposons	22,207	13,703,639	0.97
Rolling-circles	1,504	458,855	0.03
Unclassified	1,203,054	326,717,778	23.05
Small RNA	13,868	30,709,060	2.17
Satellites	128	47,021	0.00
Simple repeats	603,428	41,007,044	2.89
Low complexity	163,171	14,169,642	1.00
Total	2,465,884	1,086,657,512	76.67

Retroelements			
Type	Number of elements	Length (bp)	Coverage in genome (%)
SINES	13,160	1,678,841	0.12
LINEs	36,529	26,299,893	1.86
LTR elements—Ty1/Copia	187,929	320,865,058	22.64
LTR elements—Gypsy/DIRS1	160,992	259,764,164	18.33
LTR elements—retroviral	25,920	40,339,228	2.85
LTR elements—others	33,994	10,897,289	0.77

Repetitive elements of Hap 2			
Type	Number of elements	Length (bp)	Coverage in genome (%)
Retroelements	464,120	636,317,063	42.91
DNA transposons	26,861	16,281,690	1.10
Rolling-circles	1,781	473,989	0.03
Unclassified	1,414,803	362,236,882	24.43
Small RNA	12,182	22,910,841	1.54
Satellites	104	41,919	0.00
Simple repeats	637,863	41,654,744	2.81
Low complexity	188,519	17,334,693	1.17
Total	2,746,233	1,097,251,821	73.99

Retroelements			
Type	Number of elements	Length (bp)	Coverage in genome (%)
SINES	15,624	1,996,449	0.13
LINEs	42,506	29,505,767	1.99
LTR elements—Ty1/Copia	184,357	287,549,490	19.39
LTR elements—Gypsy/DIRS1	165,733	280,261,487	18.9
LTR elements—retroviral	17,479	24,749,986	1.67
LTR elements—others	38,391	12,253,884	0.83

The telomeric repeats of *H. sabdariffa* were analyzed using repeat masking results. The telomeric repeat sequence of *H. sabdariffa* genome was “TTTAGGG,” which is known as the canonical repeat sequence of plants (Richards and Ausubel 1988). *H. sabdariffa* has 72 telomeric regions. Among the 2,602 telomeric repeat regions in primary assembly, 67 ends of contigs were inferred to putative telomere that were positioned at the end of each contig and longer than 1,000 bp (∼140 concatemers; Fig. 2b). In Hap 1 and Hap 2, 47 and 42 ends of contigs were annotated as putative telomeres (Fig. 2b). The longest putative telomeres were 7,916 bp in primary assembly, 6,973 bp in Hap 1, and 7,916 bp in Hap 2 (Fig. 2b). The occurrence of WGD events in *H. sabdariffa* was estimated through analysis of a Ks plot (refer to Fig. 2c). A clustering method revealed the possibility of 2 or 3 instances of WGD in *H. sabdariffa*. The presence of an elevated number of genes in the primary genome assembly of *H. sabdariffa* suggests the potential occurrence of relatively recent WGD events.

We produced about 26 Gb of total RNA sequencing reads, of which 94.8% were higher than Q30 quality (Table 1). Each soft-masked genome was gene-annotated hinted by aligning those total RNA sequencing data onto each genome. A total of 154,226 genes in primary assembly, 79,143 genes in Hap 1, and 87,414 genes in Hap 2 were annotated (Table 1). The mean and N90 length of genes were 1,033 and 480 bp for primary assembly, 1,074 and 510 bp for Hap 1, and 1,099 and 525 bp for Hap 2, respectively (Table 4). They are comparable to the number of protein-coding genes, which are 87,603, and have an average CDS length of 1,188 base pairs in *H. syriacus*, implying the validity of the annotated genes. Each annotated gene set was translated into protein sequence and validated by protein BUSCO with the same database to genome BUSCO (eudicots_odb10 database). The completeness rates were 99.3% for primary assemble genome, 88.9% for Hap 1, and 92.0% for Hap 2 (Table 4). The RNA-seq read alignment rate onto the genic region of each genome was calculated by using BEDTools. A total of 94.8, 95.5, and 95.0% of RNA reads are aligned onto the genic region of the primary, Hap 1, and Hap 2 genomes, respectively.

**Table 4.** Gene annotations of *H. sabdariffa* genome.

Annotated genes of *H. sabdariffa* genome			
	Primary	Hap 1	Hap 2
Number of genes	154,226	79,143	87,414
Mean CDS (bp)	1033	1074	1099
CDS N90 (bp)	480	510	525
Protein BUSCO (eudicots_odb10)			
BUSCO completeness	99.3%	88.9%	92.0%
RNA-seq alignment rate			
Genic region	94.8%	95.5%	95.0%

### SNP and SV calling

**Fig. 3.**
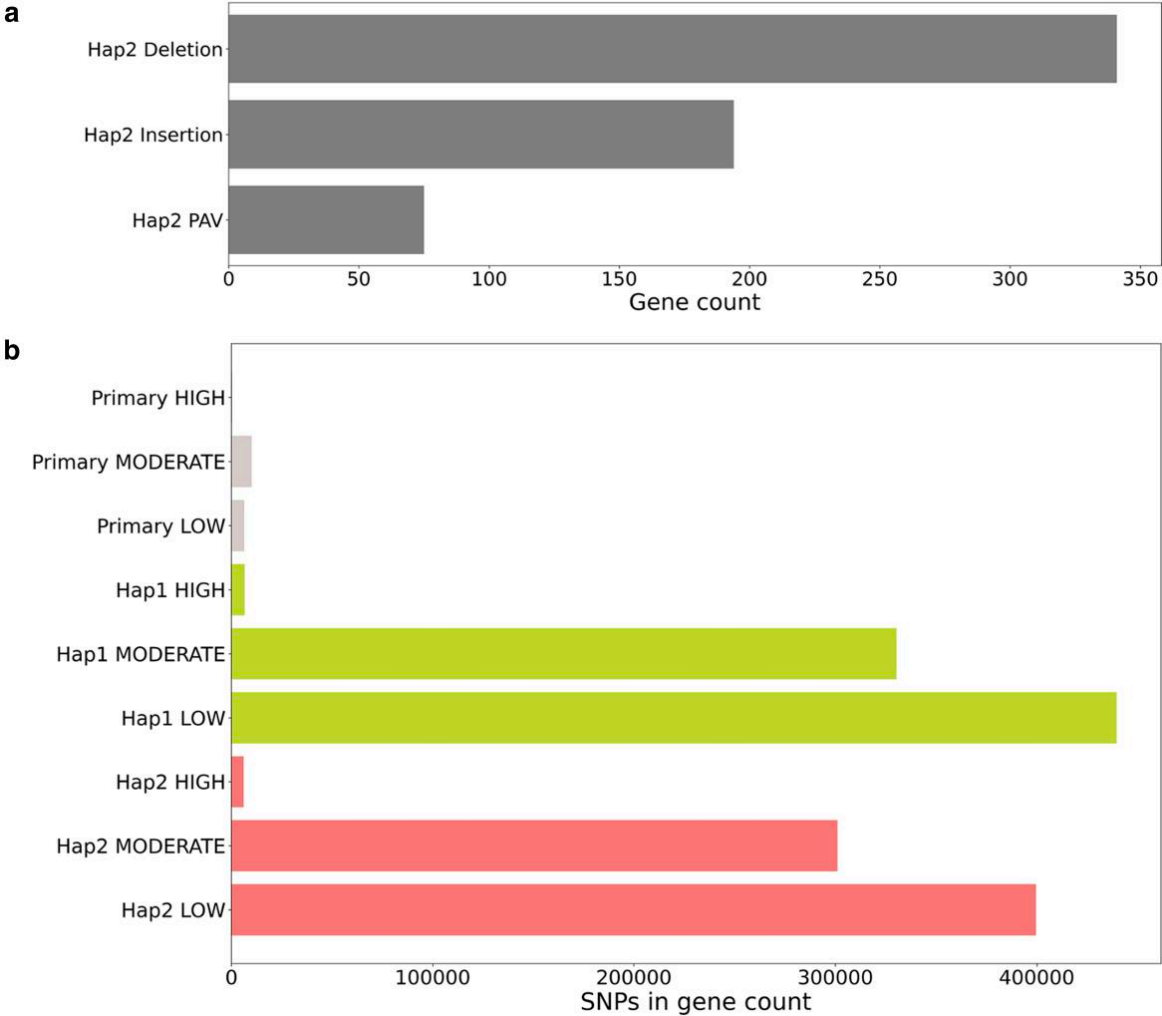
a) The number of genes related to SVs between Hap 1 and Hap 2. b) The number of SNPs related to genes in primary assembly, Hap 1, and Hap 2 genomes. The effects of SNP were classified with high, moderate, and low, which are defined by SnpEff.

SNPs in primary, Hap 1, and Hap 2 genomes were analyzed for measuring genomic variation. The impact of SNP on transcripts was classified into 3 categories: high, moderate, and low following the criteria of SnpEff (Cingolani *et al*. 2012). In the primary genome, we found 496 high-impact, 10,196 moderate-impact, and 6,147 low-impact SNPs and ∼6,000 high-impact, ∼300,000 moderate-impact, and ∼400,000 low-impact SNPs in the Hap 1 and Hap 2 genomes (Fig. 3b). Subsequently, the SVs between Hap 1 and Hap 2 genomes were called and their intersections with CDSs were analyzed to find out gene sets that were affected by these SVs. Hap 2 genome contained 341 deletions, 194 insertions, and 75 presence–absence variants on corresponding number of genes (Fig. 3a). Given the redundancy observed in tetraploid organisms, these genes might have the potential for neofunctionalization.

### Analysis of putative biosynthetic genes

**Fig. 4.**
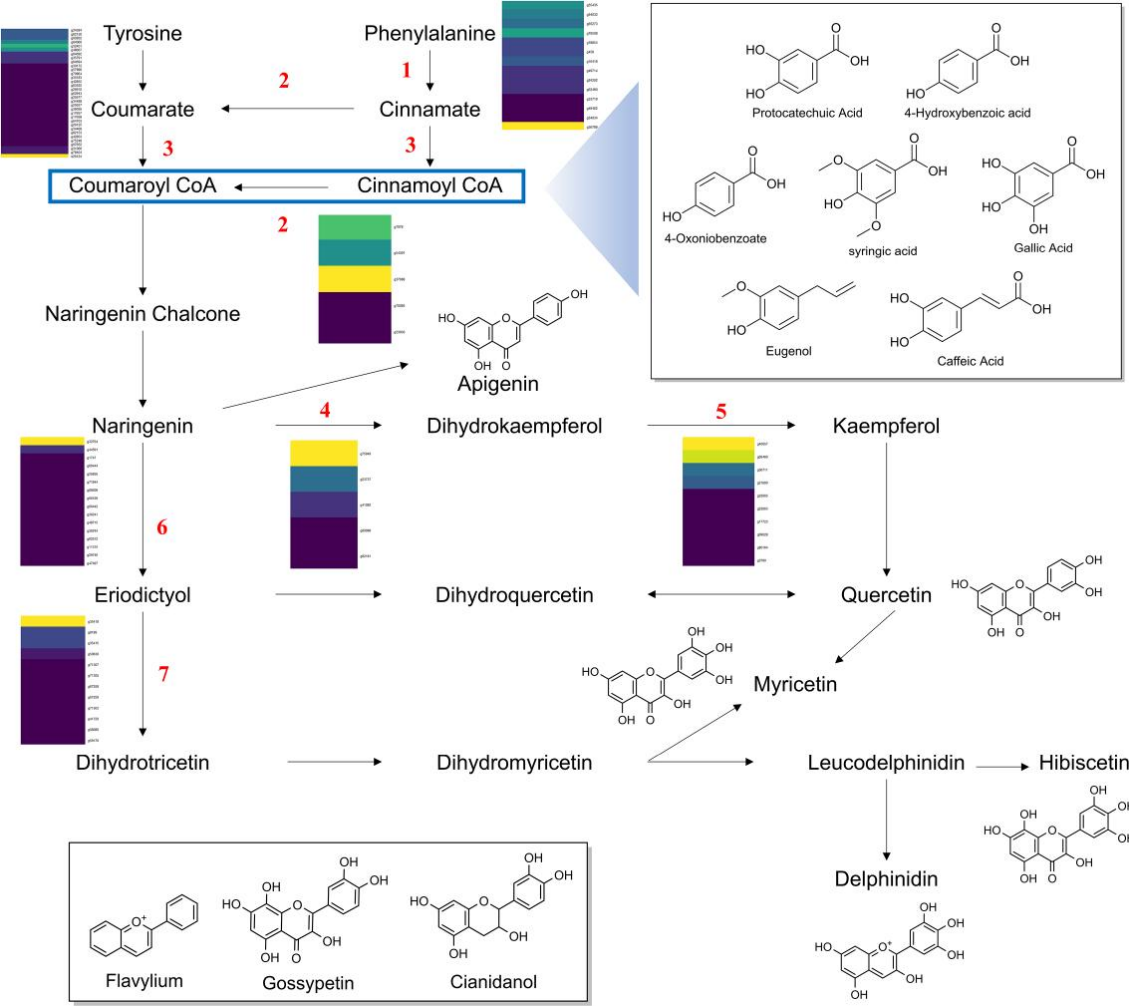
Phenylpropanoids and flavonoids of *H. sabdariffa*. The compounds in boxes are phenylpropanoids, including organic acids. The name and number of biosynthetic candidate genes in Hap 2 corresponding to each black arrow are the following: 1, phenylalanine ammonia-lyase (14); 2, trans-cinnamate 4-monooxygenase (5); 3, 4-coumarate—CoA ligase (33); 4, 2-oxoglutarate 3-dioxygenase (5); 5, flavonol synthase (10); 6, flavonoid 3′-monooxygenase (16); and 7, flavonoid 3′,5′-hydroxylase (12). The heatmaps displayed alongside numbers indicate the relative expression levels of candidate genes in RNA sequencing data, which were utilized for gene annotation. The brighter colors of genes represent higher expression.

All genes were functionally annotated based on similarity with the universal protein knowledgebase (The UniProt Consortium 2017). The taxonomic descriptions of proteins were further utilized to select candidate genes that are responsible for biosynthesis of roselle-enriched natural products: phenylpropanoids and flavonoids (Da-Costa-Rocha *et al*. 2014; Kim *et al*. 2021). The biosynthetic pathways of key intermediates involved in phenylpropanoids and flavonoids have been well characterized (Dewick 2002; Kanehisa and Goto 2000). Cinnamoyl-CoA and *p*-coumaroyl-CoA are the core frameworks of phenylpropanoids, and kaempferol and dihydrotricetin are known as the core intermediates of flavonoids. The candidate genes responsible for producing those intermediates were conserved in the *H. sabdariffa* genome (Fig. 4; Supplementary Data 2). The expression level of those candidate genes was represented by TPM in Fig. 4 and Supplementary Data 4. To understand the biosynthetic pathway of roselle-enriched natural products, the first step would involve experimentally validating the catalytic activity of candidate genes. Furthermore, those genes could be used as baits in coexpression analysis to identify uncharacterized biosynthetic genes for roselle-enriched natural products such as hibiscetin (Fig. 4; Da-Costa-Rocha *et al*. 2014; Kang *et al*. 2022).

Glycosylation stands out as a crucial modification in the diversification of natural products. Medicinal natural products found in *H. sabdariffa*, such as quercetin-*O*-glucuronide and delphinidin-3-sambubioside, are known for their functions and are synthesized through glycosylation of intermediate flavonoids (Juliani *et al*. 2009; Herranz-López *et al*. 2020). However, the process of glycosylation remains elusive in both feasible chemical synthesis and known biosynthesis pathways. In our investigation, we identified 91 genes associated with flavonoid-*O*-glycosylation and assessed their expression levels in leaves (Supplementary Data 5). Only 6 genes represented TPM higher than 5. These genes present promising candidates in the search for novel and valuable glycosylation enzymes, potentially contributing to the production of medicinal natural products. The candidate biosynthetic gene clusters that include flavonoid glycosylation have been investigated in Hap 2 genome (Supplementary Data 6).

**Fig. 5.**
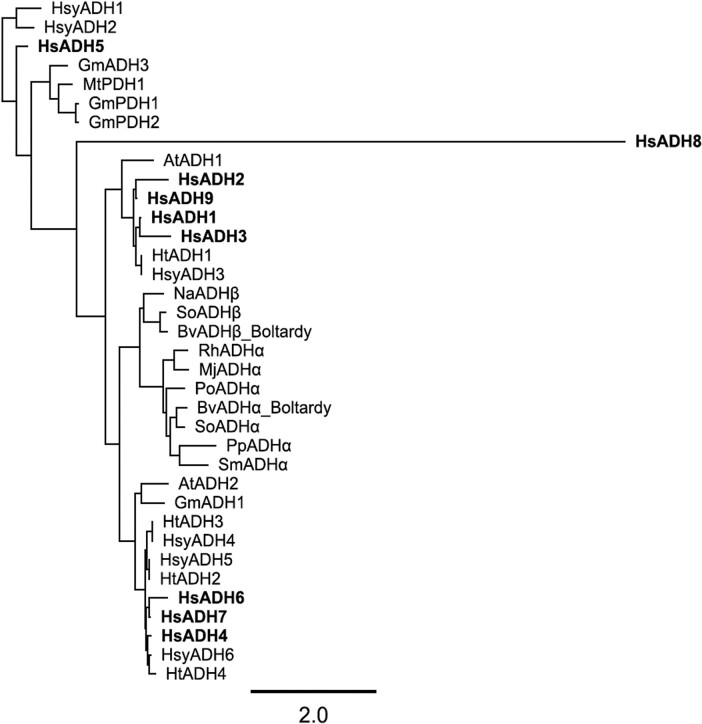
Phylogeny analysis of TyrA enzymes. Hs, *H. sabdariffa* (bold in the figure); Hsy, *H. syriacus*; Ht, *H. trionum*; Gm, *G. max*; Mt, *M. truncatula*; At, *A. thaliana*; Na, *N. alata*; So, *S. oleracea*; Bv, *B. vulgaris*; Rh, *R. humilis*; Mj, *M. jalapa*; Po, *P. oleracea*; Pp, *P. polygonifolia*; Sm, *S. marina*.

L-Tyrosine (Tyr) serve as a primary metabolite and a fundamental precursor for the phenylpropanoid and flavonoid pathways (Fig. 4). The biosynthesis of Tyr involves 2 distinct pathways: the arogenate dehydrogenase (referred to as ADH, TyrA_a_) and the prephenate dehydrogenase (referred to as PDH, TyrA_p_) pathways (Schenck *et al*. 2015). These early primary metabolism pathways, comprising the key regulatory enzymes ADH and PDH, are critical in modulating carbon flux toward Tyr biosynthesis (Schenck *et al*. 2015). This intricate pathway exhibits evolutionary variation among diverse plant lineages, necessitating thorough investigation. The predicted number of genes for encoding TyrA proteins was 10, 14, and 4 for *H. sabdariffa*, *H. syriacus*, and *H. trionum*, respectively. Employing a maximum likelihood phylogenetic analysis, the protein sequences of the candidate genes were compared with TyrA proteins from other organisms (Fig. 5; Supplementary Data 3). HsADH1–3, HsADH9, HsyADH3, and HtADH1 formed a clade with AtADH1. Similarly, HsADH4, HsADH6, HsADH7, HsyADH4–6, and HtADH2–4 clustered with AtADH2 (Fig. 5). Given the in vitro biochemical experimental validation of AtADH1 and AtADH2 (Rippert and Matringe 2002b, 2002a), these enzymes emerge as primary targets for subsequent validation as representatives of ADH activity.

MtPDH1, GmPDH1, and GmPDH2 formed a distinct clade alongside other ADHs; these enzymes exhibit PDH activity in addition to the conventional ADH activity, which has only been detected in some legumes (Schenck *et al*. 2015). HsyADH1, HsyADH2, and HsADH5 also formed unique clades with other TyrA enzymes. This indicates the need for biochemical validation of the catalytic potential of HsyADH1, HsyADH2, and HsADH5 in the production of 4-hydroxyphenylpyruvic acid from prephenate. Validating catalytic functions of these enzymes would provide crucial insights. We did not identify a gene associated with legume ADH in *H. trionum*. It is worth noting that many plants lack legume class ADH genes, which are for preventing negative feedback by phenylalanine. While our data do not provide a complete explanation, we speculate that the regulation of ADH may hold less significance in *H. trionum*, or alternatively, *H. trionum* may have evolved distinct mechanisms for upregulating the phenylpropanoid pathway. This phylogenetic approach holds promise for unraveling the biosynthetic pathway of roselle-enriched natural products within *H. sabdariffa*.

### Comparative genomics

**Fig. 6.**
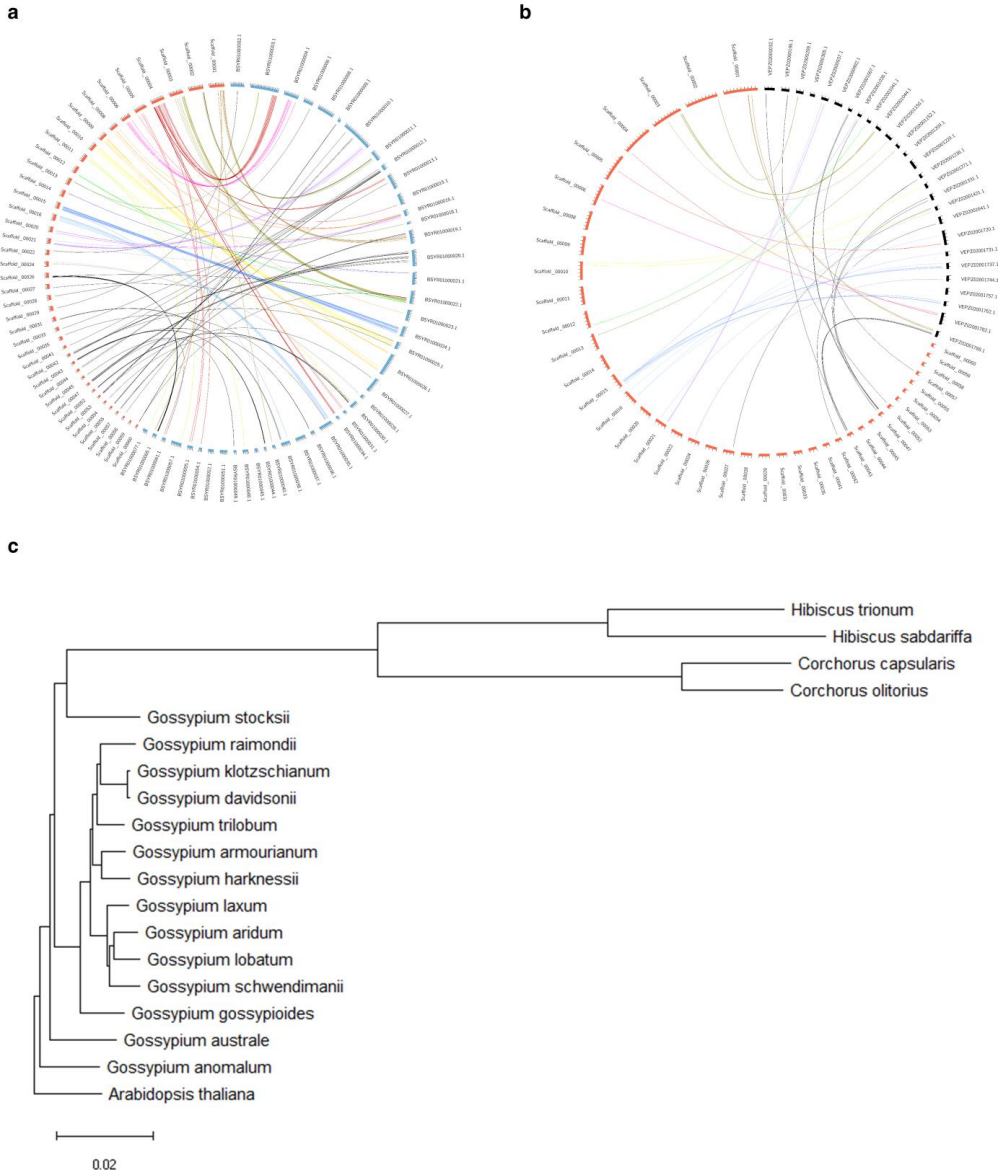
Comparative genomic analysis of the genome of *H. sabdariffa*. Genome-wide synteny analysis of the genome of *H. sabdariffa* compared with a) *H. trionum* and b) *H. syriacus*, respectively. Left of a and b, *H. sabdariffa*; right of a, *H. trionum*; and right of b, *H. syriacus*. c) Phylogenomic analysis among the Malvaceae species and *A. thaliana*.

A genome-wide synteny analysis was conducted on the genomes of *Hibiscus* genus organisms, using shared BUSCO genes (Fig. 6a and b). The resulting Circos plot illustrates the positions of shared BUSCO genes and the physical relationships between each assembled unit in *H. sabdariffa* and *H. trionum* (Fig. 6a) as well as in *H. sabdariffa* and *H. syriacus* (Fig. 6b). The linkage revealed the physical synteny between the genomes of these species. Notably, Scaffold_00004 of *H. sabdariffa* Hap 2 genome exhibited synteny with chromosome 3 of the *H. trionum* genome (Fig. 6a). These findings not only confirm the integrity of our assembled genome but also provide resources for comparative genomic analysis within the *Hibiscus* genus.

Phylogenomic analysis was carried out on the Malvaceae family and *A. thaliana*, incorporating the assembled Hap 2 genome of *H. sabdariffa*. The results demonstrated that *A. thaliana* was as an outgroup, forming a distinct clade, while the genera *Gossypium*, *Corchorus*, and *Hibiscus* formed as distinct entities in the phylogenetic tree (Fig. 6c). Noteworthy is the consistency of our findings with chloroplastic genomic sequence-based phylogenetic analyses of the *Gossypium* genus (Wu *et al*. 2018). This analysis identified a distinct genetic cluster comprising *G. anomalum*, *G. austral*, *G. stocksii*, and other *Gossypium* species, which supports our results (Fig. 6c). Additionally, *H. trionum* and *H. sabdariffa* formed a clade, and *C. capsularis* and *C. olitorius* constituted another clade, both distinct from the *Gossypium* clade (Fig. 6c). The phylogenomic analysis could serve as a foundational resource for comparative genomics within the Malvaceae family.

### Conclusion

Roselle is an important medicinal plant producing diverse natural products. However, the lack of genomic resource had limited the biological understanding of roselle and its pharmacological applications. In this study, we report the high-quality genome of *H. sabdariffa* and putative genes related with its metabolism. Our results could be a foundation for understanding genetic evolution and metabolism in roselle.

## Supplementary Material

jkae134_Supplementary_Data

## Data Availability

Genome assembly and associated data are available on National Center for Biotechnology Information (NCBI): primary genome assembly and annotation (PRJNA1077781), Hap 1 genome assembly and annotation (PRJNA1077780), Hap 2 genome assembly and annotation (PRJNA1077779), RNA-seq data (PRJNA1072585), and genome sequencing data (PRJNA1013732). Supplemental material available at G3 online.
